# Transcriptomic analyses on muscle tissues of *Litopenaeus vannamei* provide the first profile insight into the response to low temperature stress

**DOI:** 10.1371/journal.pone.0178604

**Published:** 2017-06-02

**Authors:** Wen Huang, Chunhua Ren, Hongmei Li, Da Huo, Yanhong Wang, Xiao Jiang, Yushun Tian, Peng Luo, Ting Chen, Chaoqun Hu

**Affiliations:** 1 CAS Key Laboratory of Tropical Marine Bio-resources and Ecology (LMB), South China Sea Institute of Oceanology, Chinese Academy of Sciences, Guangzhou, Guangdong, China; 2 Key Laboratory of Applied Marine Biology of Guangdong Province and Chinese Academy of Sciences (LAMB), South China Sea Institute of Oceanology, Chinese Academy of Sciences, Guangzhou, Guangdong, China; 3 South China Sea Bio-Resource Exploitation and Utilization Collaborative Innovation Center, Guangzhou, Guangdong, China; 4 University of the Chinese Academy of Sciences, Beijing, China; Chang Gung University, TAIWAN

## Abstract

The Pacific white shrimp (*Litopenaeus vannamei*) is an important cultured crustacean species worldwide. However, little is known about the molecular mechanism of this species involved in the response to cold stress. In this study, four separate RNA-Seq libraries of *L*. *vannamei* were generated from 13°C stress and control temperature. Total 29,662 of Unigenes and overall of 19,619 annotated genes were obtained. Three comparisons were carried out among the four libraries, in which 72 of the top 20% of differentially-expressed genes were obtained, 15 GO and 5 KEGG temperature-sensitive pathways were fished out. Catalytic activity (GO: 0003824) and Metabolic pathways (ko01100) were the most annotated GO and KEGG pathways in response to cold stress, respectively. In addition, Calcium, MAPK cascade, Transcription factor and Serine/threonine-protein kinase signal pathway were picked out and clustered. Serine/threonine-protein kinase signal pathway might play more important roles in cold adaptation, while other three signal pathway were not widely transcribed. Our results had summarized the differentially-expressed genes and suggested the major important signaling pathways and related genes. These findings provide the first profile insight into the molecular basis of *L*. *vannamei* response to cold stress.

## Introduction

The Pacific white shrimp, *Litopenaeus vannamei*, is one of the most important cultural shrimp species, and its production (by weight) has reached nearly 71% of the total economic penaeid shrimp production worldwide [1, 2, 3, 4]. Naturally, *L*. *vannamei* can inhabit estuaries, lagoons or marine areas, but they are sensitive to low temperatures [1]. With high economic value, the farming areas of *L*. *vannamei* have been exploded from the southern to the northern coast, and to the inland water in China. Therefore, low temperatures have become one of the major constrained factors to t *L*. *vannamei* culture, since that the culturing water temperature of the Northern China in winter is usually lower than that needed for *L*. *vannamei* survival.

Water temperature is one of the most common abiotic stress factors for aquatic ectotherms [5]. It influences nearly all biochemical and physiological progresses of aquatic organisms [6], and low temperatures may cause severe growth inhibition and mortality [6, 7]. In fishes, the understanding of biochemical and molecular mechanisms under temperature stress have been well developed. In species such as rainbow trout (*Oncorhynchus mykiss*) [8], channel catfish (*Ictalurus punctatus*) [9] and common carp (*Cyprinus carpio L*.) [10], studies showed that about 10 percent of the investigated genes were found to be up-regulated under temperature stress, and they were involved in an extensive gene expression network [11]. Digital gene expression in Nile tilapia (*Oreochromis niloticus*) revealed the differentially expressed-genes when comparing the fish cultured in 10°C and 30°C [12]. These studies provided useful insights and promoted the molecular breeding work in fish species. However, in *L*. *vannamei*, the gene expression profile in response to temperature stress has not yet been illustrated.

RNA-Seq technology is a crucial method for quantifying the transcriptional expression in non-model species or in those organisms that lack whole-genome sequencing data [13, 14]. Studies on channel catfish (*I*. *punctatus*) have suggested that both the de novo synthesis of cold-induced proteins and the modification of existing proteins were required for adaptation to low temperatures [9]. Transcriptional regulation research in the coral reef fish *Pomacentrus moluccensis* revealed the existence of differentially expressed genes in this species [15]. Moreover, RNA-Seq has already been applied as a useful method on other stress response studies in *L*. *vannamei*, such as high salinity [16] and low salinity [17, 18, 19, 20]. However, the specific expression profile report on *L*. *vannamei* under the stress of cold temperature is still lacked.

Molecular signaling pathways can help to improve the understanding of the activity and coordination of the cell-expression network in response to extracellular stress. Calcium is a ubiquitous second messenger in eukaryotic signal transduction cascades [21, 22]. It impacts nearly every aspect of cellular life [23] and fulfills its key role in transferring extracellular stress signals into chromatin and in regulating protein phosphorylation [24]. Structural comparisons of mammalian Ca^2+^/calmodulin-dependent protein kinase II (CaMKII) and plant calcium-dependent protein kinases (CDPKs) of calcium signaling showed that their kinase domain containing the highly conserved subdomains of typical eukaryotic Ser/Thr protein kinases [22]. Mitogen-activated protein kinases (MAPKs), regulated by MAPK kinase kinase-MAPK kinase-MAPK (MKKK-MKK-MAPK) phosphorelay system, are Ser/Thr protein kinases and are key components for vital signal transduction pathways that converting extracellular stimuli into a wide range of cellular responses in the organisms from yeast to humans [25,26,27]. Consequently, in order to understand the molecular basis of *L*. *vannamei* under cold temperatures, investigations conducted on differentially expressed genes in signaling were necessary.

Shrimps are poikilothermic species, for which changing of water temperatures can conduct to each part of their bodies and influence their cellular activities. Muscle compose nearly 60% body weight of *L*. *vannamei*, and it is the main tissue for converting biochemical energy in shrimp [6]. Furthermore, we found that the muscle tissues of *L*. *vannamei* turned whitish immediately when they were chilled in low temperature water, but regained pellucidity after being returned to the control temperature, indicating that the muscle tissues of *L*. *vannamei* may process overt physiology activities in response to acute cold stress. In the present study, a transcriptomic analysis has been carried out on the muscle of *L*. *vannamei*. Gene expression profiles under the stress of low temperature has been characterized by comparing various libraries from corresponding temperature treatments, including acute cold stress for 2 hours, endurance cold stress for 48 hours and recovering to normal temperature for 2 hours. The identified candidate genes will be useful and helpful in revealing a deeper insight into the molecular adaptation basis under low temperature stress in *L*. *vannamei*.

## Materials and methods

### Ethics statement

Shrimp care and experiments were carried out according to the Care and Use of Agricultural Animals in Agricultural Research and Teaching and approved by the Science and Technology Bureau of China. Approval from the Department of Wildlife Administration was not required for the experiments conducted in this paper. All experiments in this paper were performed with permits obtained from the Government of the People’s Republic of China and endorsed by the Animal Experimentation Ethics Committee of Chinese Academy of Sciences.

### Treatments and cold challenges of the shrimp materials

Healthy shrimp with an average weight of 7.09±3.22 g were treated in gradient temperatures (9, 10, 11, 12, 13, 14, 15, 18, 21, 24, 27, 30 and 33°C) for 2 hours, 48 hours and then recovered at a control temperature (28°C) for 2 hours. Survival was evaluated by observing the shrimp’s ability to move spontaneously or after gentle prodding [28]. The temperature of semi-death group was set as the stress cold temperature.

Thirteen degree was set as the stress cold temperature. Four groups were placed either in control temperature (CT), 13°C for 2 hours (T_2), 13°C for 48 hours (T_48) and then, the T_48 group was placed to recover temperature for 2 hours (RC). Muscle tissues from the surviving individuals were put in centrifuge tubes and dipped into liquid nitrogen immediately. Three biological replicates in equal amounts of each group (2 μg of RNA for each individual, and total 6 μg of RNA for each group) were pooled for subsequent RNA-Seq experiments.

### RNA isolation and cDNA library construction

Four separate libraries (CT, T_2, T_48 and RC) were sampled. Total RNA was extracted by TRIzol Reagent (Invitrogen, USA) according to the manufacturer’s instructions. The quality of the RNA products was verified as described by Ren et al. (2014) [14]; RNase-free DNase I (Invitrogen, USA) was used to degrade any possible DNA, and oligo (dT)-coated magnetic beads were mixed with the total RNA to concentrate the ployA tailed mRNA. Fragmentation was performed by incubation in an NEB Next First Strand Synthesis Reaction Buffer (NEB, USA), and the second strand was generated with the buffer, dNTPs, RNase H and DNA polymerase-I. Adapters were ligated to the synthesized cDNA fragments after an end repair step.

### RNA-Seq and annotation

The library was constructed using an Illumina HiSeq 4000 sequencing platform located at the BGI Company (Shenzhen, China; http://www.genomics.cn/index). The clean read data were deposited to the US National Center for Biotechnology Information (NCBI) Sequence Read Archive (SRA, http://www.ncbi.nlm.nih.gov/Traces/sra) with the accession No. SRP095377.

After assembling, functional annotation by gene ontology terms (GO, http://www.geneontology.org) was analyzed using the Blast2GO program [29]. The Kyoto Encyclopedia of Genes and Genomes Pathway (KEGG; http://www.genome.jp/kegg), the Non-redundant NCBI collection of nucleotide (NT) and protein (NR) sequence database (ftp://ftp.ncbi.nih.gov/blast/db/FASTA/), the Swiss-Prot database (http://web.expasy.org/docs/swiss-prot_guideline.html) and the Cluster of Orthologous Groups (COG) protein database (http://www.ncbi.nlm.nih.gov/COG/) were used to predict and classify the possible functions [30].

### Quantification of differentially expressed genes

Three comparisons were taken to the four libraries, including CT-VS-T_2 (T_2/CT), T_2-VS-T_48 (T_48/T_2), and T_48-VS-RC (RC/T_48). The differentially expressed genes (DEGs) in comparison of T_2/CT represented the transcripts in response to acute cold stress (placed from control temperature to acute 13°C). The DEGs in comparison of T_48/T_2 represented the transcripts in response to endurance cold stress (endured the cold stress for 48 hours). The DEGs in comparison of RC/T_48 represented the transcripts for recover adaptation (placed from stress cold to control temperature). The clean reads per kilobase per million (RPKM) value was used to estimate transcript abundance on the basis of eliminating the influence of different gene lengths and sequencing discrepancies [31]. Therefore, the RPKM values could be directly used to compare the differences of gene expression among samples. The algorithm developed by Audic and Claverie (1997) [32] was used to compare the differences in various gene expression levels. A value of the FDR (false discovery rate) of less than 0.001 and a value of the Log2 ratio of less than 1 were set as the criteria values.

### Identification of major response genes and pathways

The coexisted DEGs in different comparisons of total genes, GO annotated genes and KEGG annotated genes were filtered. Four important kinds of signaling genes regarding Calcium and MAPK cascades, Transcription factor and Serine/threonine-protein kinase signaling genes were searched from the total annotated genes [14]. To understand the expression model of target genes, hierarchical clustering analysis was performed with the software Heatmap Illustrator Heml 1.0.3.3.

### Real-time PCR validation

To verify the expression profiles of genes in our RNA-Seq results, the represented 15 DEGs in comparison of T_2/CT were selected for validation of the Illumina sequences by real-time PCR analysis. All primers used were listed in Table 1. The treatments of *L*. *vannamei*, sampling of muscle, isolation of total RNA and treatment of DNase were performed as described above. The first cDNA strand was synthesized by PrimeScript® Reverse Transcriptase Kit (Takara, China) with an oligo (dT) primer, a qPCR analysis was conducted in a RotorGene RG3000 real-time PCR system (Corbett research, Australia), PCR reactions were conducted using a SYBR Premix Ex TaqTM II (Takara, China), and the reactions were exposed to an initial denaturation (94°C for 3 min) followed by 40 cycles of 94°C for 25 s, 60°C for 15 s, and 72°C for 15 s. The relative transcript abundance was obtained by normalizing with expression of the *L*. *vannamei β-actin* gene based on the 2^-ΔΔCT^ method [33].

**Table 1:** The primers used for Real-time PCR.

Gene Name	GeneID	Product length	Primers
TN-C	Unigene7411_All	105 bp	F: GCTACATCACCACCGATACCC R: ACCCGTCTTCGTCCACCT
E74	Unigene9779_All	150 bp	F: ACTTGTGGGAGTTCCTTCTG R: TGTCGGGCTTGTTCTTGT
MLP	Unigene3723_All	94 bp	F: CAGGAAGCCGTGAAGAAG R: AGAGGACGACTTGAGGAAT
MLC3	Unigene9994_All	88 bp	F: CGCAGACAGACAGCCATGT R: CGAACACATTGCTTCCTCCTC
SBK1-like	Unigene11894_All	109 bp	F: CTATGGAGACCTGTCGCAGTT R: GTCCTTGCTGTGAACGAACTC
Ago 4	Unigene25375_All	135 bp	F: CAACATAGACCTGGCTCATACG R: CCACATTCCAATCGCTTCCTTA
IGF2BP	Unigene5734_All	102 bp	F: AGGTTGGACGCATAATTGGTAA R: CTGAAGTTGTGGTTCCTTGTTG
AP1B1	CL1921.Contig2_All	93 bp	F: TGGGCGGTGGATTAGATT R: AGGATGTGGCAGTTGGAG
TIA1-like	CL1624.Contig5_All	79 bp	F: GAAAGATGCGAGTTGATT R: TGATGATGCCTACTGGTG
DUSP3	CL199.Contig2_All	130 bp	F: GCAGCAGGGAGGAAAGGT R: CTTCTTGCTGATGGTGGC
FreD	Unigene15069_All	119 bp	F: ACGCCAGCACCAAGTATCG R: GCCGTCGTTGTCGTTGTCA
SERPIN	Unigene5290_All	99 bp	F: GAACGCCGCCTACTTCAAG R: ACGAGGTCGCTGTTCTGAG
PMCA	Unigene14415_All	98 bp	F: CGCAGTCAGTCATTGTCAGTC R: CAACAGCCATTAGCCACTACC
VLG	Unigene26017_All	130 bp	F: AACAACAGAAGGCAAGACAACT R: CGGATGATTCAGTAGCACCAA
HSP 70	CL261.Contig3_All	103 bp	F: TTGTGAACGAGCGAAGAGGA R: GGCACGAGTGACAGAGGTATA
β-actin	GeneBank: AF300705	121 bp	F: GCCCATCTACGAGGGATA R: GGTGGTCGTGAAGGTGTAA

## Results

### Cold stress temperature

**Fig 1:**
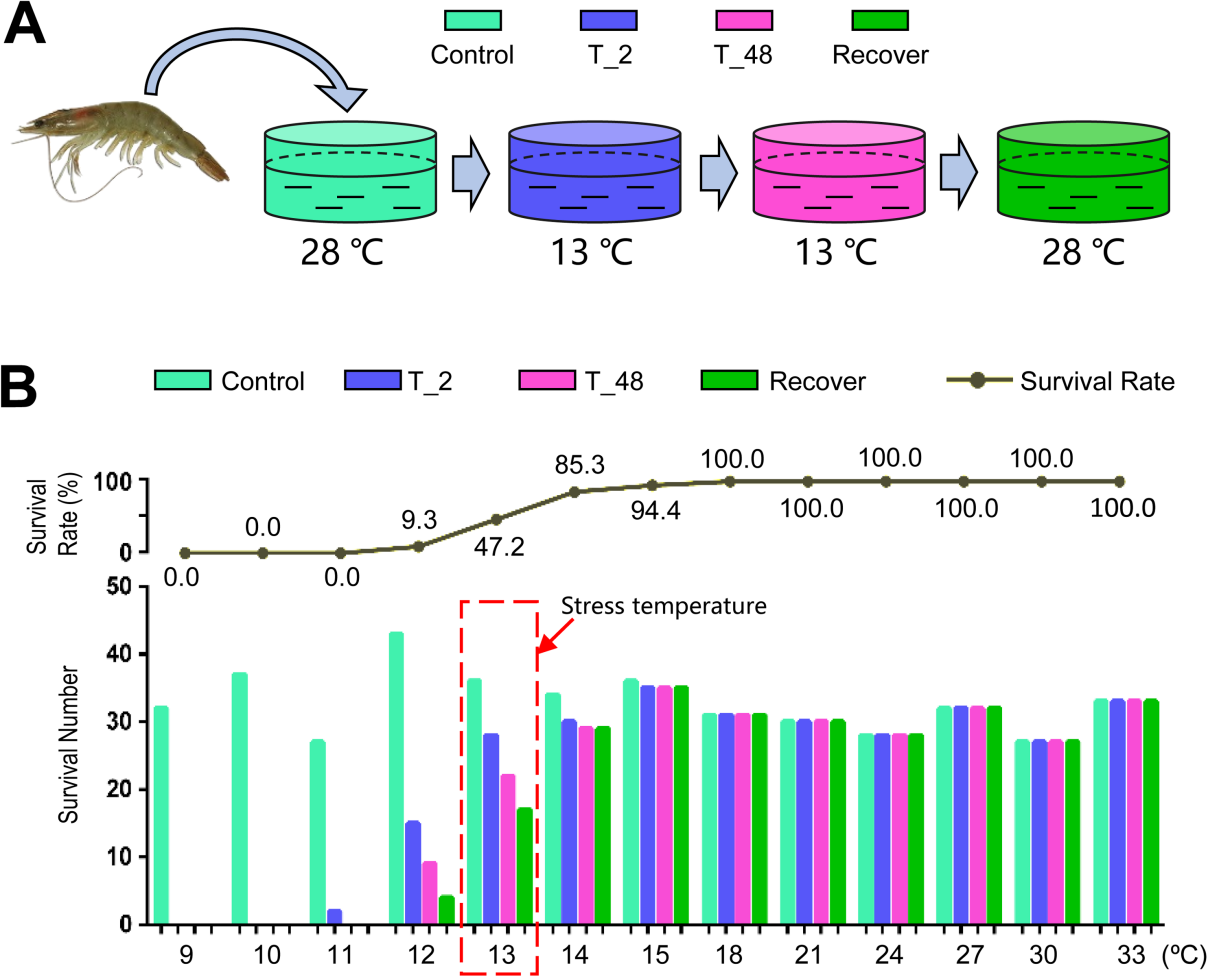
The cold challenge and survival rate of *L*. *vannamei*. (A): Treatment processes of the shrimp. (B): Gradient temperature challenge and survival rates. Control: treated in control temperature of 28℃; T_2: treated in related temperature for 2 hours; T_48: treated in related temperature for 48 hours; Recover: treated in control temperature of 28℃ for 2 hours recovering. Red frame indicated the stress temperature.

A batch of *L*. *vannamei* were treated with different temperatures, and the survival rates were summarized (Fig 1B). The results showed that *L*. *vannamei* could not survive when treated with 11°C or lower temperatures, the survival rates were slightly affected in the water at 14°C, 15°C or higher temperatures (Fig 1B). The survival rates at 12°C and 13°C were 9.30% and 47.22%, respectively. We used 13°C as the cold stress temperature since a nearly half survival rate (47.22%) of shrimp was observed at this temperature. The same batch of shrimp from the same cultural environment was used for the following experiments.

### Overview of the libraries

**Fig 2:**
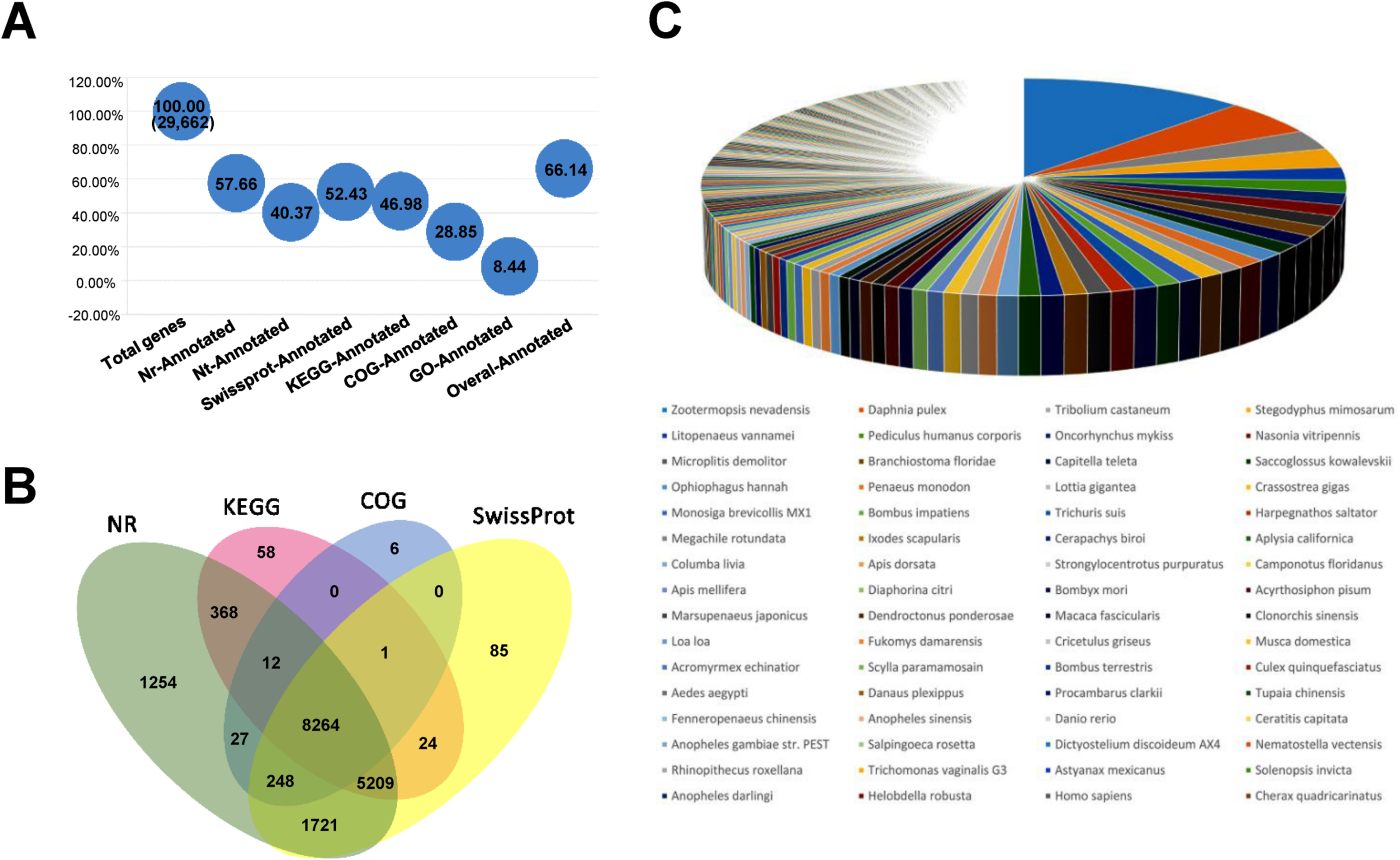
Annotation of the transcriptome. **(**A): The summary results with main annotation database; the X axis were labeled with various items; Y axis represented the proportion genes annotated by the functional database; 29,662 was the total numbers of Unigenes, only the percentage numbers were marked in other blue dots. (B): The Venn diagram between NR, KEGG, COG and Swissprot; the annotated gene numbers were exhibit in the related areas. (C): The pie chart of percentage of annotated species with NR database; the main species names were displayed under the pie chart, species (with the corresponding color) were displayed with descending order.

After filtering, the quality metrics of the reads are showed in Table 2: the total raw reads of the samples were from 87.69 to 90.94 Mb, the number of clean reads per library ranged from 65.23 to 66.02 Mb, and the clean reads ratio ranged from 71.95% to 75.28%. The mean value of clean reads Q20 and Q30 percentages were 97.41% and 94.34%, respectively. The clean reads were assembled and clustered into Unigenes, and the quality metrics of Unigenes are presented in Table 3. The total number of All-Unigenes was 29,662, the total length was 29,632,338 bp and the mean length was 999 bp. The annotated genes in NR, NT, SwissProt, KEGG, COG, GO database were 17,103 (57.66%), 11,976 (40.37%), 15,552 (52.43%), 13,936 (46.98%), 8,558 (28.85%) and 2,504 (8.44%), respectively (Fig 2A). Overall 19,619 Unigenes were annotated (Fig 2A). The main Venn diagram of NR, KEGG, COG and SwissProt annotation are showed in Fig 2B, the largest interior gene numbers were 8,264. The percentage of annotated species with NR database are displayed in Fig 2C, the top three annotated species were *Zootermopsis nevadensis* (the largest blue part, 12.34%), *Daphnia pulex* (the second large tenne part, 5.31%) and *Tribolium castaneum* (the third large gray part, 2.95%), respectively.

**Table 2:** Summary of quality metrics reads after filtering.

Sample	Total Raw Reads (Mb)	Total Clean Reads (Mb)	Total Clean Bases (Gb)	Clean Reads Q20^a^ (%)	Clean Reads Q30^b^ (%)	Clean Reads Ratio (%)
CT	90.94	65.43	9.81	97.43	94.38	71.95
T_2	90.94	65.54	9.83	97.37	94.28	72.07
T-48	87.69	66.02	9.90	97.40	94.31	75.28
RC	87.69	65.23	9.78	97.44	94.38	74.38

^a ^Q20: the rate of bases whitch quality is greater than 20

^b^ Q30: The rate of bases which quality is greater than 30

**Table 3:** Quality metrics of Unigenes.

Sample	Total Number	Total Length	Mean Length	N50^a^	N70^b^	N90^c^	GC(%)^d^
CT	25734	20408154	793	1472	688	293	44.84
T_2	26759	21470873	802	1481	705	295	44.42
T_48	23679	18917663	798	1515	699	291	44.77
RC	25885	21579584	833	1625	745	297	44.83
All-Unigene	29662	29632338	999	2148	1099	334	44.84

^a^ N50: a weighted median statistic that 50% of the TotalLength is contained in Unigenes great than or equal to this value

^b^ N70: a weighted median statistic that 70% of the TotalLength is contained in Unigenes great than or equal to this value

^c^ N90: a weighted median statistic that 90% of the TotalLength is contained in Unigenes great than or equal to this value.

^d^ GC (%): the percentage of G and C bases in all Unigenes

### GO and KEGG classification of the DEGs

GO annotation was conducted for the three comparisons. In the comparison group of T_2/CT, 870 of 1471 DEGs could be assigned by GO classification, and the equivalent numbers for other comparison groups were 1147 of 1943 DEGs (T_48/T_2) and 690 of 1477 DEGs (RC/T_48); the detailed GO classification data (Pathway, PathwayID, and GeneID) are listed in S1 Table. The most annotated GO pathway in each comparison was Catalytic activity (GO: 0003824).

**Fig 3:**
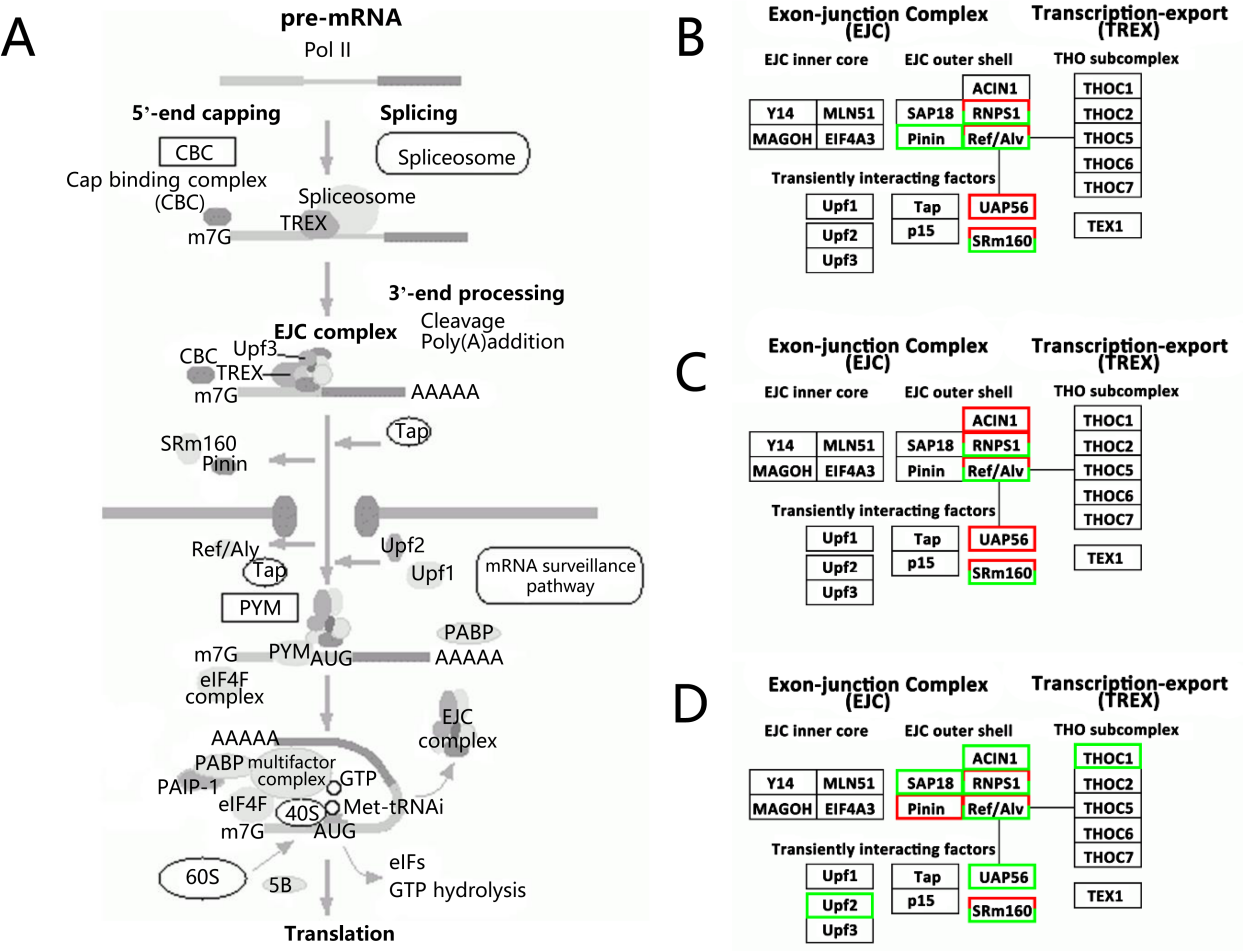
The main components and DEGs in RNA transport pathways. (A): mRNA translation processes. (B): the annotated different expressed genes in EJC complex of T_2/CT. (C): the annotated different expressed genes in EJC complex of T_48/T_2. (D): the annotated different expressed genes in EJC complex of RC/T_48. The up-regulated genes were marked with red frame, down-regulated genes were marked with green frame.

KEGG annotation was conducted for the three comparisons. In the comparison of T_2/CT, 1202 of 1471 DEGs were assigned to 83 pathways. In the comparison of T_48/T_2, 1556 of 1943 DEGs were assigned to 91 pathways. In the comparison of RC/T_48, 1208 of 1477 DEGs were assigned to 77 pathways. The details of the KEGG classification (Pathway, PathwayID, and GeneID) of the three comparisons are listed in S2 Table. The top two annotated KEGG pathways in all the three comparisons were Metabolic pathways (ko01100) and RNA transport pathways (ko03013). The RNA transport pathway maps (there were no Metabolic pathway maps in KEGG database) were traced and downloaded from the KEGG database (S1 Fig), the annotated DEGs in the three comparisons were summarized (S3 Table), the major discrepant DEGs in RNA transport pathway were the components of exon-junction complex (EJC). The annotated up- and down-regulated genes of EJC complex were marked and displayed in Fig 3. The EJC complex mainly participated in mRNA translation (S1 Fig, Fig 3A), the EJC outer shell and Transiently interacting factor genes were the most two kinds of responding components in EJC complex (S3 Table, Fig 3B, 3C and 3D).

**Fig 4:**
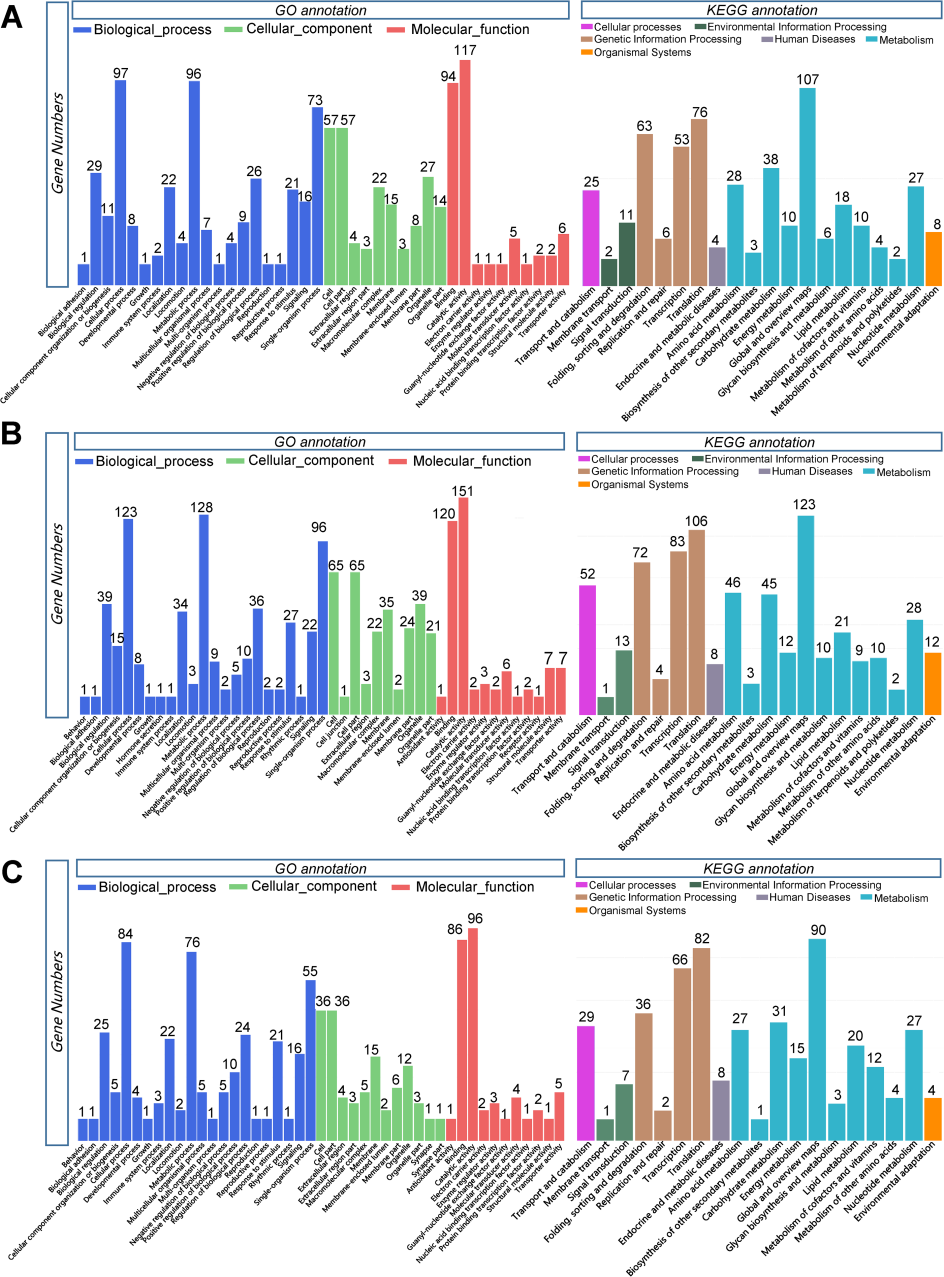
GO and KEGG classification of the DEGs in each comparison. (A): Comparison of T_2/CT. (B): Comparison of T_48/T_2. (C): Comparison of RC/T_48. The Y-axis represents the number of DEGs in each item.

Represented GO and KEGG classification columnar figures in different comparisons are showed in Fig 4. GO classes contained three categories, including Biological processes, Cellular components and Molecular functions. The top two annotated classes of Biological processes in the comparison of T_2/CT were Cellular processes (97 annotated genes) and Metabolic processes (96 annotated genes), the major classes of Cellular components were Cell (57 annotated genes) and Cell parts (57 annotated genes), and the major classes of Molecular functions were Binding (94 annotated genes) and Catalytic activity (117 annotated genes). The top two annotated KEGG classes in comparison of T_2/CT were Global and overview maps (107 annotated genes of Metabolism) and Translation (76 annotated genes of Genetic Information Processing). The most annotated GO classes in comparison of T_48/T_2 were Catalytic activity (151 annotated genes) in Molecular function, Cell (65 annotated genes) and Cell junction (65 annotated genes) in Cellular component, Metabolic process (128 annotated genes) in Biological process; the most top two annotated KEGG classes were Global and overview maps (123 annotated genes of Metabolism) and Translation (106 annotated genes of Genetic Information Processing). The most annotated GO classes in comparison of RC/T_48 were Catalytic activity (96 annotated genes) in Molecular function, Cell (36 annotated genes) and Cell part (36 annotated genes) in Cellular component, Cellular process (84 annotated genes) in Biological process; the most top two KEGG classes were Global and overview maps (90 annotated genes of Metabolism) and Translation (82 annotated genes of Genetic Information Processing).

### Quantification of transcripts

**Fig 5:**
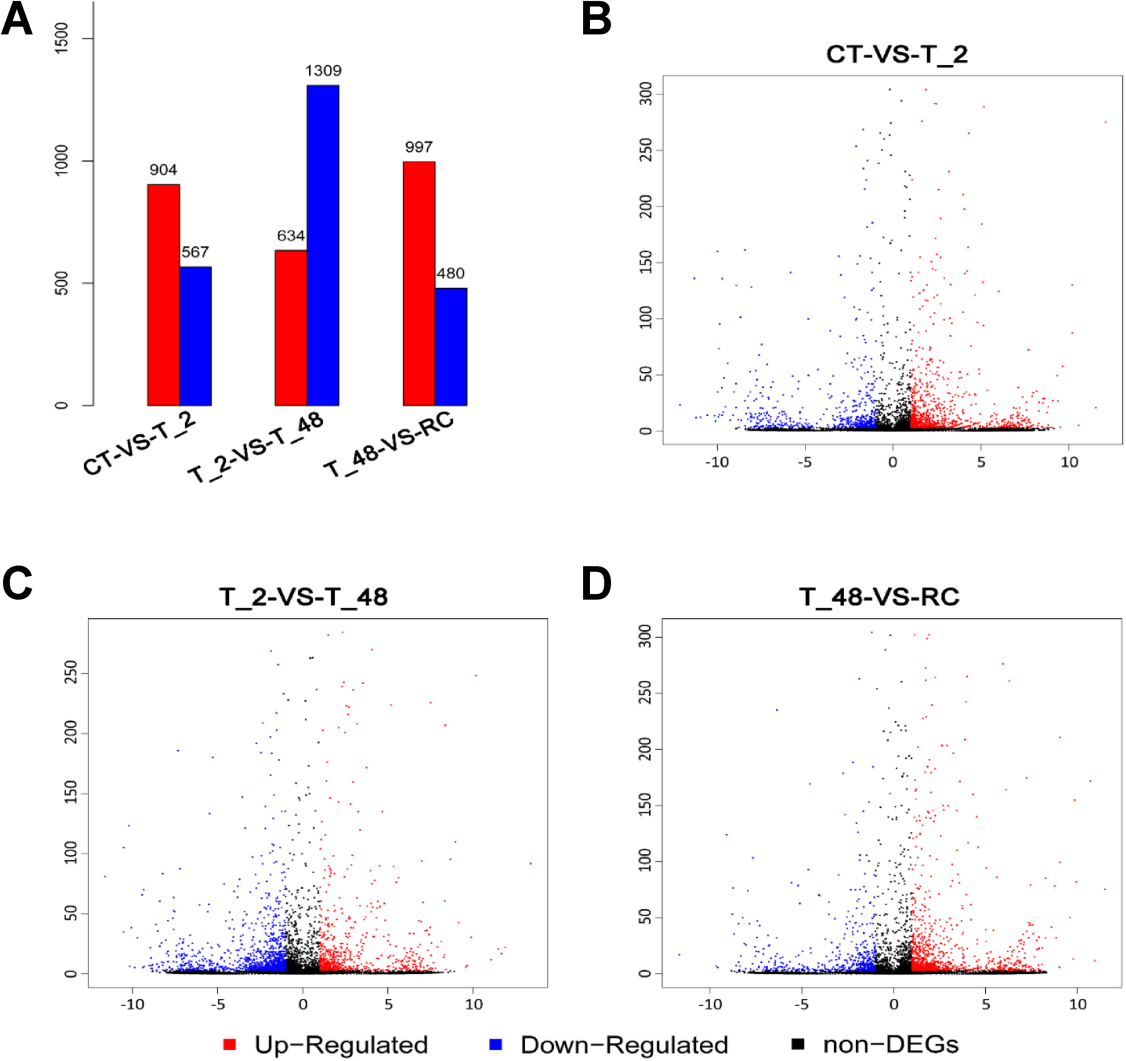
Column and Volcano-plot of the DEGs in comparisons. The columnar results of the DEGs are listed and the corresponding Volcano-plot figures are shown. Plots in the Volcano-plot figures represent the DEGs. The Y-axis in the columnar figures represents the DEG numbers in comparisons. In the Volcano-plot figures, the X-axis represents the comparison value of log_2_^FoldChange^, and the Y-axis represents the value of |-log_10_^FDR^|. The red color refers to up-regulated DEGs, blue color refers to down-regulated DEGs, and the black plot refers to no significantly different DEGs between the pairs of libraries (FDR value ≤ 0.001, |log_2_^FoldChange^| value ≥ 1).

The transcript abundance of each Unigene from the four related libraries is listed in S4 Table. DEGs were identified through a pairwise comparison between various transcripts by setting a threshold FDR (false discovery rate) value of 0.001 and a |log_2_^FoldChange^| value of 1 based on an algorithm described by Audic and Claverie (1997) and Ren et al. (2014). From the three comparisons (T_2/CT, T_48/T_2 and RC/T_48), a large number of DEGs were identified, and the filtered results (P value < 0.05, FDR ≤ 0.001, and estimated absolute |log_2_^FoldChange^| ≥ 1) are exhibited in S5–S7 Tables. The columnar and Volcano-plot results of the DEGs are showed in Fig 5. The X-axis of Volcano-plot represented the comparison value of log_2_^FoldChange^, the Y-axis represented value of |-log_10_^FDR^|. From the pictures, CT-vs-T_2 (T_2/CT) had 904 up- and 567 down-regulated genes, T_2-vs-T_48 (T_48/T_2) had 634 up- and 1309 down-regulated genes and T_48-vs-RC (RC/T_48) had 997 up- and 480 down-regulated genes. The down-regulated transcripts were far more than the up-regulated genes in comparison of T_48/T_2, indicating that more genes were suppressed under the endurance cold stress.

### Identification of major response genes

**Fig 6:**
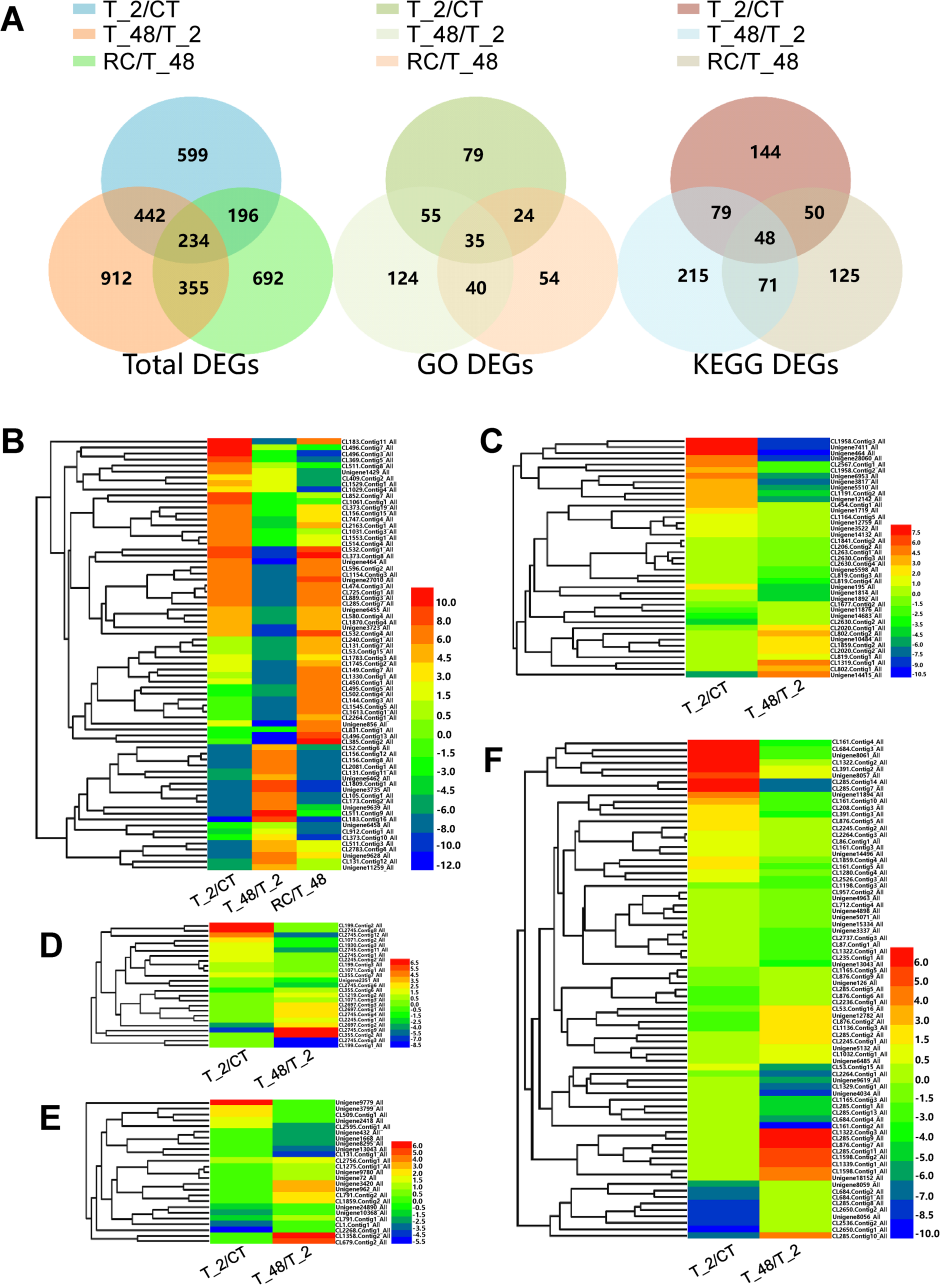
Venn diagrams and heat maps of the major coexisted DEGs. (A): The Venn diagrams of Total DEGs, GO annotated DEGs and KEGG annotated DEGs in the three comparisons; DEG numbers were labeled in the corresponding areas. (B): Hierarchical clustering analysis of the top 20% of Total coexisting DEGs in the three comparisons. (C): Hierarchical clustering analysis of the annotated Calcium signaling genes in cold stress. (D): Hierarchical clustering analysis of the annotated MAPK cascades genes in cold stress. (E): Hierarchical clustering analysis of the annotated Transcription factor genes in cold stress. (F): Hierarchical clustering analysis of the annotated Serine/threonine-protein kinase genes in cold stress.

The Venn diagrams in Fig 6A were used to exhibit the coexisted DEGs in different comparisons. It showed that 234 genes were coexisting in the three comparisons of Total DEGs, and 35 of coexisted GO annotated DEGs and 48 of coexisted KEGG annotated DEGs were also identified.

Top 20% of differently-expressed coexisted genes in the three comparisons were selected and taken to Hierarchical clustering analysis, 72 genes were obtained, the detailed GeneID and other information are showed in S8 Table, the heat map figures are showed in Fig 6B. Take the Unigene of CL532.Contig1_All as an example, significantly up-regulated (log_2_^FoldChange^ value = 9.33) in comparison of T_2/CT, down-regulated (log_2_^FoldChange^ value = -9.33) in comparison of T_48/T_2 and up-regulated (log_2_^FoldChange^ value = 8.60) in comparison of RC/T_48. The results indicated that this gene was sensitive to acute cold temperature and recover temperature, and it was down-regulated under the endurance cold stress environment. This gene might play important role in adaptation of changing temperature but was greatly inhibited in long time cold stress environment. The Unigene of Unigene3735_All was taken as another example, for which was significantly down-regulated (Log_2_^FoldChange^ Value = -7.63) in comparison of T_2/CT, up-regulated (Log_2_^FoldChange^ Value = 8.12) in comparison of T_48/T_2 and down-regulated (log_2_^FoldChange^ Value = -8.12) in comparison of RC/T_48. The results indicated this gene was transcriptional inhibited under the environment of changing temperature but was highly expressed under the environment of endurance cold stress, suggesting that it might play important role in response to long time cold stress.

### Identification of major pathways

Total 35 coexisted GO-annotated DEGs and 48 coexisted KEGG-annotated DEGs were selected, the detailed GeneID information are showed in S9 and S10 Tables. The major (Absolute Value of log_2_^FoldChange^ ≥ 5.0) Unigene in GO and KEGG Pathways were filtered, and 15 GO pathways and 5 KEGG pathways were recognized as the temperature-sensitive pathways in response to cold stress (Table 4). The Unigene of CL596.Contig2_All, described as Eukaryotic translation initiation factor 5 gene (gi|646705355|), was annotated by Translation initiation factor 5 pathway (KEGG: K03262) and Cellular macromolecule metabolic (GO: 0044260) and primary metabolic process (GO: 0044238), implying that this gene might play important role in several molecular functions in response to cold stress.

**Table 4:** The major temperature-sensitive pathways.

Pathway	GeneID	T_2/CT^a^	T_48/T_2^b^	RC/T_48^c^	Targeted GeneID and Description	Annotated pathway	PathwayID
GO pathway	CL596.Contig2_All	7.91	-7.91	7.60	gi|646705355|Eukaryotic translation initiation factor 5	Cellular macromolecule metabolic process; primary metabolic process	GO:0044260; GO:0044238
	CL474.Contig3_All	7.098	-7.098	6.392	gi|170064960|Prolyl 4-hydroxylase subunit alpha-1	Oxidoreductase activity, acting on paired donors, with incorporation or reduction of molecular oxygen, 2-oxoglutarate as one donor, and incorporation of one atom each of oxygen into both donors; ion binding	GO:0016706; GO:0043167
	CL285.Contig7_All	6.375	-6.375	6.229	gi|380028691|Serine/threonine-protein kinase MARK2-like	Phosphorylation; protein serine/threonine kinase activity; nucleotide binding	GO:0016310; GO:0004674; GO:0000166
	CL580.Contig4_All	5.907	-5.9069	5.492	gi|256574617|GTP binding protein	GTP catabolic process; adenylate cyclase-modulating G-protein coupled receptor signaling pathway; G-protein beta/gamma-subunit complex binding; GTP binding; signal transducer activity; GTPase activity	GO:0006184; GO:0007188; GO:0031683; GO:0005525; GO:0004871; GO:0003924
	CL156.Contig8_All	-6.989	6.45943	-6.46	gi|195397962|Drosophila virilis	Organic cyclic compound binding; heterocyclic compound binding	GO:0097159; GO:1901363
KEGG pathway	CL596.Contig2_All	7.91	-7.91	7.60	gi|646705355|Eukaryotic translation initiation factor 5	Translation initiation factor 5	K03262
	Unigene464_All	7.70	-10.50	6.41	gi|356713486|Troponin C2	Troponin C, skeletal muscle	K12042
	CL474.Contig3_All	7.10	-7.10	6.39	gi|170064960|Prolyl 4-hydroxylase subunit alpha-1	Prolyl 4-hydroxylase	K00472
	CL1870.Contig4_All	5.32	-5.32	5.67	gi|646719569|Protein transport protein Sec31A	Protein transport protein SEC31	K14005
	CL2081.Contig1_All	-7.29	6.09	-6.09	gi|665801408|Uncharacterized protein LOC103572085 isoform X1	THO complex subunit 4	K12881

^a ^T_2/CT: The value of Log_2_^FoldChange^ in comparison of T_2/CT

^b ^T_48/T_2: The value of Log_2_^FoldChange^ in comparison of T_48/T_2

^c ^RC/T_48: The value of Log_2_^FoldChange^ in comparison of RC/T_48

### Major signaling genes in response to cold stress

Four important kinds of signaling genes regarding Calcium signaling, MAPK cascades, Transcription factors and Serine/threonine-protein kinase were fished out (S11–S14 Tables) and clustered into heat map. Generally, the Calcium signaling genes, MAPK cascades genes and Transcription factor genes were not widely transcribed in response to cold stress (Fig 6C, 6D and 6E). Four Unigenes of Calcium signaling genes, 4 Unigenes of MAPK cascades genes and 5 Unigenes of Transcription factor genes were obtained when filtered by the top two levels of log_2_^FoldChange^ (absolute value of log_2_^FoldChange^ ≥ 6.0 in Calcium signaling genes, absolute value of log_2_^FoldChange^ ≥ 5.0 in MAPK cascades genes and absolute value of log_2_^FoldChange^ ≥ 3.5 in Transcription factor genes, Table 5). Whereas, conspicuous patches of Serine/threonine-protein kinase genes were observed (Fig 6F), 16 Unigenes were obtained when filtered by the top two levels of log_2_^FoldChange^ (absolute value of log_2_^FoldChange^ ≥ 5.0). The results suggested that Serine/threonine-protein kinase might participate in the adaptation of acute or endurance cold stress.

**Table 5:** Major signaling genes in response to cold stress.

Signal Category	GeneID	T_2/CT^a^	T_48/T_2^b^	Targeted GeneID	Gene description
Ca	Unigene7411_All	8.22	-8.22	gi|298106306|	Troponin C isoform 3
	CL1958.Contig3_All	8.83	-8.83	gi|378947923|	Sarco/endoplasmic reticulum Ca2+-ATPase
	Unigene28060_All	5.62	-8.95	gi|383860510|	Troponin C, isoform 2-like
	Unigene464_All	7.70	-10.50	gi|356713486|	Troponin C2
Mapk	CL199.Contig2_All	7.67	0.00	gi|241638524|	Dual specificty phosphatase, putative
	CL2745.Contig8_All	7.19	0.00	gi|646715586|	C-jun-amino-terminal kinase-interacting protein 3
	CL2745.Contig9_All	-5.88	7.29	gi|646715586|	C-jun-amino-terminal kinase-interacting protein 3
	CL199.Contig1_All	0.00	-7.98	gi|241638524|	Dual specificty phosphatase, putative
Ser/Thr	CL1329.Contig1_All	0.00	-6.25	gi|242023108|	Serine/threonine-protein kinase prp4, putative
	CL285.Contig7_All	6.38	-6.38	gi|646709938|	MAP/microtubule affinity-regulating kinase 3, partial
	CL285.Contig14_All	6.48	-6.48	gi|662183391|	Serine/threonine-protein kinase par-1-like
	CL2264.Contig1_All	-1.14	-6.51	gi|321472272|	Hypothetical protein DAPPUDRAFT_315683
	Unigene4034_All	0.00	-7.39	gi|675380112|	Serine/threonine-protein kinase VRK1, partial
	CL161.Contig2_All	0.00	-9.43	gi|646695066|	Serine/threonine-protein kinase Doa
	CL1322.Contig3_All	0.00	6.87	gi|307189141|	Serine/threonine-protein phosphatase 2A 65 kDa regulatory subunit A alpha isoform
	CL285.Contig9_All	0.00	6.75	gi|646709938|	MAP/microtubule affinity-regulating kinase 3, partial
	CL285.Contig8_All	-7.44	0.00	gi|321475182|	Hypothetical protein DAPPUDRAFT_313175
	CL2650.Contig2_All	-7.62	0.00	gi|312083017|	Hypothetical protein LOAG_08105
	Unigene8056_All	-7.81	0.00	gi|646704391|	Serine/threonine-protein kinase VRK1
	CL2536.Contig2_All	-8.44	0.00	gi|669328025|	Hypothetical protein M514_19703
	CL2650.Contig1_All	-9.92	0.00	gi|312083017|	Hypothetical protein LOAG_08105
	CL161.Contig4_All	7.58	-3.88	gi|646695066|	Serine/threonine-protein kinase Doa
	CL1322.Contig2_All	7.09	0.00	gi|307189141|	Serine/threonine-protein phosphatase 2A 65 kDa regulatory subunit A alpha isoform
	CL684.Contig3_All	7.09	-1.60	gi|573876090|	Arginine/serine-rich protein PNISR-like
TFs	CL1358.Contig2_All	0.00	7.13	gi|665809417|	Nucleosome-remodeling factor subunit NURF301 isoform X3
	CL679.Contig2_All	0.00	5.75	gi|321464944|	Putative transcriptional factor Homothorax protein
	CL131.Contig1_All	0.00	-3.98	gi|332031390|	Protein daughterless
	Unigene9779_All	6.55	0.00	gi|645003508|	Ecdysone-induced protein 74EF-like isoform X1
	CL2268.Contig1_All	-5.49	0.00	gi|645003508|	Ecdysone-induced protein 74EF-like isoform X1

^a ^T_2/CT: The value of Log_2_^FoldChange^ in comparison of T_2/CT

^b^ T_48/T_2: The value of Log_2_^FoldChange^ in comparison of T_48/T_2. Ca: Calcium signaling pathway genes Mapk: Mitogen-activated protein kinases cascades genes; Ser/Thr: Serine/threonine-protein kinase genes; TFs: Transcription factor genes

### Real-time PCR validation

**Fig 7:**
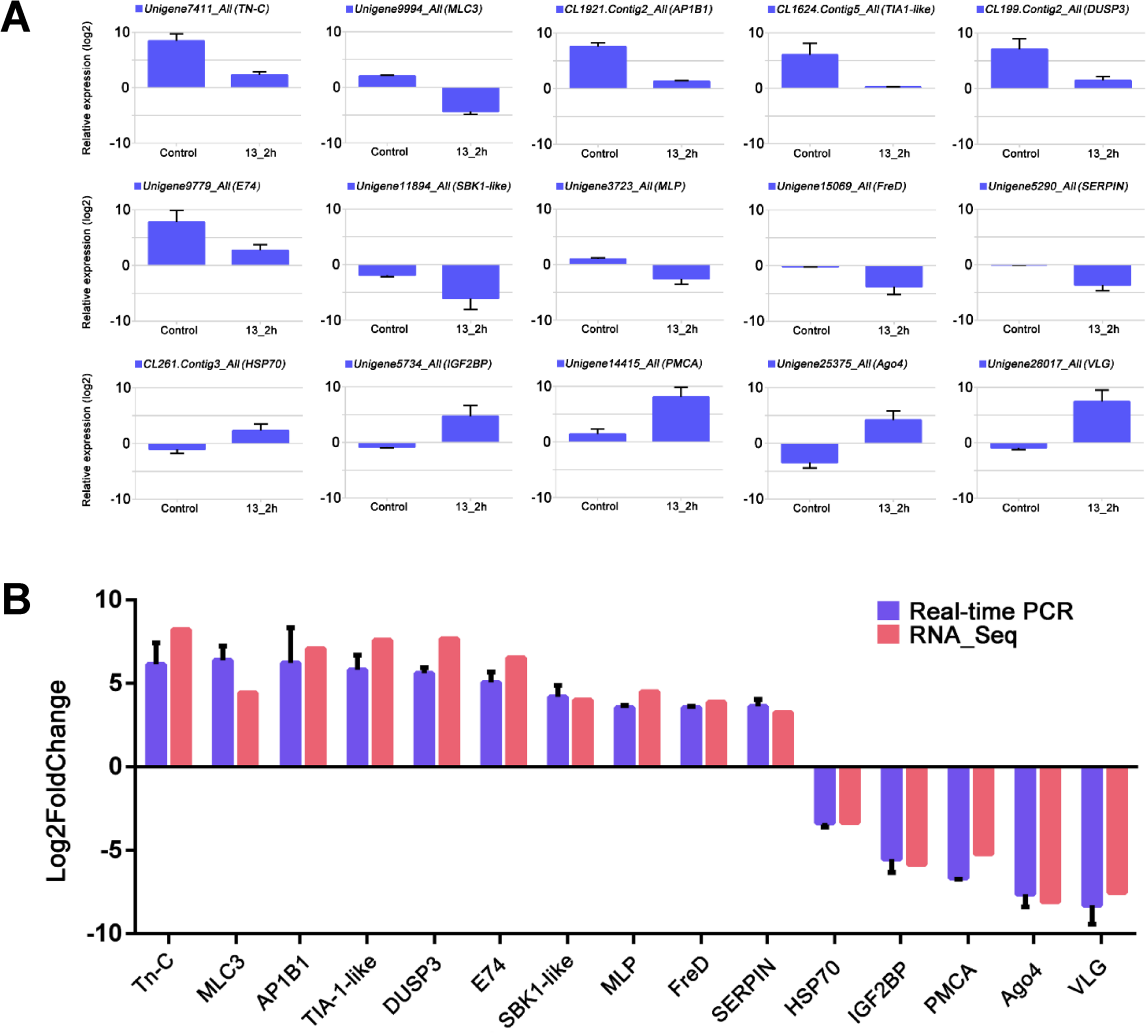
Validation for the 15 transcriptomic DEGs in the comparison of T_2/CT. (A): The Real-time PCR results of the 15 genes in treatments of CT and T_2; Y axis represented the Log2 value of the related expression. (B): Grouped results of the 15 genes; the blue columnar mean the Real-time PCR results, red columnar mean the RNA-Seq results; X axis indicated the gene names, Y axis represented the value of log_2_^FoldChange^ value in the comparison of T_2/CT, the FoldChange value were obtained by2^–ΔΔCT^ method. Bars in this figure represented the standard deviation (SD).

The Real-time PCR outcomes were further summarized. The qPCR results of the 15 genes in the treatments of CT and T_2 are showed in Fig 7A. Grouped comparison results between qPCR and RNA-Seq were also displayed, and the results showed all the 15 candidate genes in qPCR verification consisted with the results of the RNA-Seq technology (Fig 7B).

## Discussion

Temperature is one of the most important environmental variances for aquatic ectotherms, and it has been investigated for its key role in affecting overall biological processes in various species [7, 34]. In larval zebrafish (*Danio rerio*), cold stress induced-transcripts were generated from 2 and 48 hours treatments under 16°C [11]. In tilapia (*O*. *niloticus*), an analysis of gene expressions was carried out under cold stress by comparing different temperatures, from 30°C followed by a decrement of 1°C/Day to 10°C [12]. In Manila clam (*Ruditapes philippinarum*), a transcriptome study was conducted in response to cold stress with an extreme low temperature of -1°C [35]. Stresses could be classified into mild, chronic or acute, which represented a dramatic shift in the environmental conditions [36]. In our study, shrimp were treated in acute cold temperatures for 2 hours, 48 hours and then in a recovery temperature for 2 hours, and a nearly 50% survival rate (47.22%) was observed in the 13°C group. The stress temperature in our result was similar to that reported by Fan et al. (2013) [37], in which 13°C for 24 hours was regarded as a suitable cold stress temperature to investigate the altered proteins and genes in *L*. *vannamei*.

RNA-Seq technology has been demonstrated as a straightforward method to reveal the general molecular regulation processes in species underlying the response to stressful environments [14]. In the previous studies for *L*. *vannamei*, RNA-Seq technology has been employed to investigate the different gene expression levels involved in various environment factors and developmental processes, such as a transcriptome analysis under acute ammonia stress [38], some digital gene expression analyses in response to low salinity stress [17, 18, 19, 20, 39], and the transcriptional profiling during ontogenesis [40]. Parts of the molecular mechanisms have already been characterized in response to temperature in the shrimp. The protein content of CAT, GST, Ferritin and HSP60 have been found to be influenced by temperature stress in *L*. *vannamei* [41]. Lipid peroxidation, catalase activity and glutathione-S-transferase in *Palaemon elegans* and *Palaemon serratus* have been proved as the biomarkers for monitoring aquatic environments [42]. In addition, the decreasing water temperatures has been shown to induce oxidative stress in *L*. *vannamei* [43, 44]. Furthermore, 20 proteomic spots and several genes have demonstrated to play important roles in temperature regulation of *L*. *vannamei* [37]. However, the profile information of the regulation of gene expression under low temperatures in *L*. *vannamei* was still lacked. In the present study, transcriptomic analyses were conducted under cold temperature stress on the muscle of *L*. *vannamei*. To our knowledge, this is the first report of transcriptional profiling under acute low temperature stress in *L*. *vannamei*.

Transcriptome analyses may provide insights into the molecular basis of cold tolerance in many species. Transcriptomic characterization under temperature stress in zebrafish (*D*. *rerio*) revealed the transcriptional and post-transcriptional regulation of the cold-acclimated processes [5], the multiple temperature-regulated biological processes and pathways [11], and the over-expressed biological processes (such as circadian rhythm, energy metabolism, lipid transport and metabolism) [19]. An RNA-Seq analysis in *Melanotaenia duboulayi* provided candidate genes in response to changing temperatures in freshwater fish [45]. The study of transcriptomic analysis on the marine fish ectoparasitic ciliate *Cryptocaryon irritans* under a low temperature revealed the required genes for cell survival and a deeper dormancy state [46]. In our present study, a transcriptomic analysis for *L*. *vannamei* under acute low temperature was carried out. In total of the four libraries, 904 up- and 567 down-regulated genes in comparison of T_2/CT, 634 up- and 1309 down-regulated genes in comparisons of T_48/T_2, and 997 up- and 480 down-regulated genes in comparisons of RC/T_48 were detected. The top 20% of coexisted DEGs were fished out. In our result, 15 GO pathways and 5 KEGG pathways were annotated as temperature-sensitive pathways. Our study promoted the understanding of profiling gene expression of *L*. *vannamei* in response to cold stress.

Acute stress places cells in danger, and rapid adaptation is crucial for maximizing cell survival [36]. To adapt to either biotic or abiotic stress, cellular signaling pathways may coordinate multiple integrated and regulated layers of different steps of mRNA biogenesis in inner cells [24, 36]. Thus, the important signaling pathways regarding cellular stress response adaptation have been investigated in many studies. Calcium ions (Ca^2+^) impact nearly every aspect of cellular life [23]; Teets et al. (2013) demonstrated that goldenrod gall fly (*Eurosta solidaginis*) tissues used calcium signaling to instantly detect decreases in temperature and to trigger downstream cold-hardening mechanisms [47]. In the transcriptomic analysis reported by Ren et al. (2014), the gene members of the calcium signaling, including cation/calcium exchangers, calcium-binding proteins, calmodulin-like proteins, etc., were found to be involved in the response to cold stress in *Chrysanthemum morifolium* [14]. The MAPKs influenced the cellular signal transduction in organisms from yeast to human. When yeast under osmostress, the Ste11 MAP3Ks were activated, followed by activation of Pbs2 MAP2K that combined to Hog1 and then phosphorylation of the specific osmostress transcription factors for cell adaption [36]. The p38 family in mammalian species contains 4 isoforms (MAPK11-14) with many overlapping functions in cells, and downstream targets of p38 MAPKs include several kinases that are involved in the control of gene expression and nuclear proteins, such as transcription factors (CREB, ATF1, NFκB, STAT1 and STAT3) and regulators of chromatin remodeling (H3 and HMG14) [36]. In this paper, the expression model for the genes in four signal pathways were investigated under cold stress. The major differentially-expressed signaling genes were suggested involve in the adaptation of cold stress. Serine/threonine-protein kinase genes might play more important roles in response to cold stress, while Calcium, MAPK cascades and Transcription factor signaling genes were not widely transcribed in response to cold stress.

In conclusion, the overall transcriptional expression and quantification analyses under the cold stress temperature of 13°C were conducted for *Litopenaeus vannamei*. Top 20% of differentially expressed genes were filtered. The most annotated and temperature-sensitive of GO and KEGG pathways were found out. The major differentially-expressed genes in signaling were suggested. The results showed that Ser/Thr kinase signal pathway might play more important roles in participating the cold adaptation, while Calcium, MAPK cascades and Transcription factors signaling genes were not widely transcribed. Our study provided the first insight into the molecular basis and supplied an important reference for the mechanism of *L*. *vannamei* responded to cold stress.

## Supporting information

S1 Fig The RNA transport pathway map from KEGG database (map03013). (TIF)

S1 Table GO classification of DEGs in each comparison. (XLSX)

S2 Table KEGG pathway classification of the DEGs in each comparison. (XLSX)

S3 Table Summary of DEGs of the main components of RNA transport pathway. (XLSX)

S4 Table The transcription level of each unigene derived from the number of relevant reads of the four libraries. The “GeneLength” column gives the length of exon sequence. "-" represented absence of this unigene in the related group of transcript. RPKM: clean reads per kilobase per million for each unigene in each libraries, respectively.
(XLSX)

S5 Table Different transcribed genes between the comparison of T_2/CT. ^a^ The “GeneLength” column gives the length of exon sequence. ^b^ CT: reads per kb per million reads (RPKM) for each unigene in transcription of Control. ^c^ T_2: reads per kb per million reads (RPKM) for each unigene in transcription of T_2. ^d^ Log2FoldChange(T_2/CT): the ratio between the RPKM in CT to the RPKM in T_2. The criteria applied for assigning significance were: Pvalue < 0.05, FDR ≤ 0.001, and estimated absolute |log2FoldChange| ≥ 1. Genes listed in descending order of absolute |log2FoldChange|. NA: No annotation were found in the related database.
(XLSX)

S6 Table Different transcribed genes between the comparison of T_48/T_2. ^a^ The “GeneLength” column gives the length of exon sequence. ^b^ T_2: reads per kb per million reads (RPKM) for each unigene in transcription of T_2. ^c^ T_48: reads per kb per million reads (RPKM) for each unigene in transcription of T_48. ^d^ Log2FoldChange(T_48/T_2): the ratio between the RPKM in T_2 to the RPKM in T_48. The criteria applied for assigning significance were: Pvalue < 0.05, FDR ≤ 0.001, and estimated absolute |log2FoldChange| ≥ 1. Genes listed in descending order of absolute |log2FoldChange|. NA: No annotation were found in the related database.
(XLSX)

S7 Table Different transcribed genes between the comparison of RC/T_48. ^a^ The “GeneLength” column gives the length of exon sequence. ^b^ T_48: reads per kb per million reads (RPKM) for each unigene in transcription of T_48. ^c^ RC: reads per kb per million reads (RPKM) for each unigene in transcription of RC. ^d^ Log2FoldChange(RC/T_48): the ratio between the RPKM in T_48 to the RPKM in RC. The criteria applied for assigning significance were: Pvalue < 0.05, FDR ≤ 0.001, and estimated absolute |log2FoldChange| ≥ 1. Genes listed in descending order of absolute |log2FoldChange|. NA: No annotation were found in the related database.
(XLSX)

S8 Table Top 20% of coexisted DEGs in the three comparisons. ^a^ T_2/CT: The value of Log2FoldChange in comparison of T_2/CT; ^b^ T_48/T_2: The value of Log2FoldChange in comparison of T_48/T_2; ^c^ RC/T_48: The value of Log2FoldChange in comparison of RC/T_48.
(XLSX)

S9 Table The coexisted DEGs in GO annotation. ^a^ T_2/CT: The value of Log2FoldChange in comparison of T_2/CT; ^b^ T_48/T_2: The value of Log2FoldChange in comparison of T_48/T_2; ^c^ RC/T_48: The value of Log2FoldChange in comparison of RC/T_48.
(XLSX)

S10 Table The coexisted DEGs of KEGG annotation. ^a^ T_2/CT: The value of Log2FoldChange in comparison of T_2/CT; ^b^ T_48/T_2: The value of Log2FoldChange in comparison of T_48/T_2; ^c^ RC/T_48: The value of Log2FoldChange in comparison of RC/T_48.
(XLSX)

S11 Table The differential gene expression of Calcium signaling genes in each comparison. (XLSX)

S12 Table The differential gene expression of MAPK cascades genes in each comparison. (XLSX)

S13 Table The differential gene expression of Transcription factors (TFs) in each comparison. (XLSX)

S14 Table The differential gene expression of Serine/threonine-protein kinase genes in each comparison. (XLSX)
