# Supplementary figures and images for: Transcriptomic analyses on muscle tissues of Litopenaeus vannamei provide the first profile insight into the response to low temperature stress
Source: PLoS One. 2017 Jun 2;12(6):e0178604. doi: 10.1371/journal.pone.0178604 (PMC5456072; doi:10.1371/journal.pone.0178604)

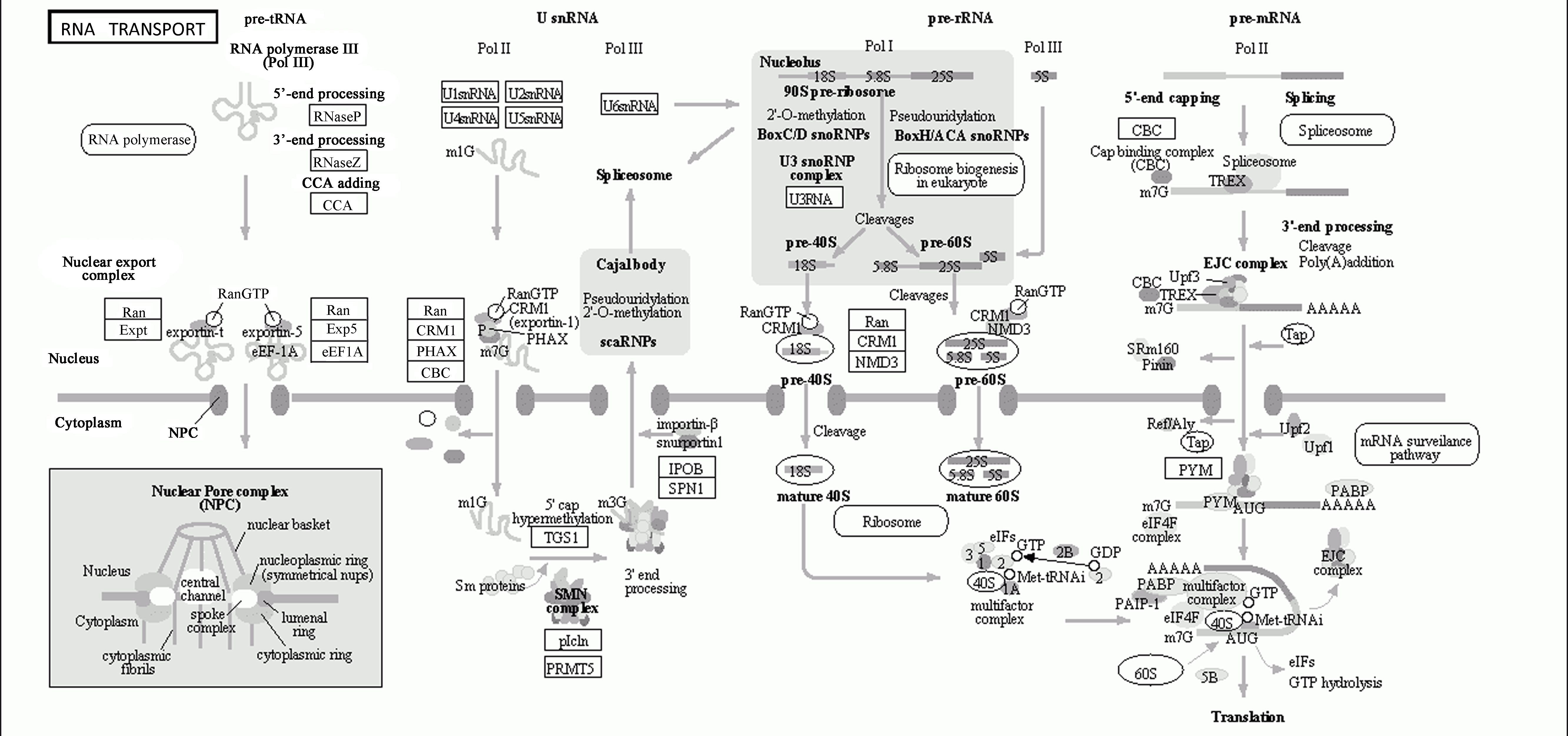

Supplement: S1 Fig — (TIF) [file pone.0178604.s001.tif]
